# Icariin inhibits chondrocyte apoptosis and angiogenesis by regulating the TDP‐43 signaling pathway

**DOI:** 10.1002/mgg3.586

**Published:** 2019-02-07

**Authors:** He Huang, Zhao‐Fei Zhang, Feng‐Wei Qin, Wang Tang, Dong‐Hua Liu, Pei‐Yu Wu, Feng Jiao

**Affiliations:** ^1^ Department of Orthopedic Surgery Guangzhou Hospital of Integrated Traditional and Western Medicine Guangzhou China

**Keywords:** apoptosis, icariin, osteoarthritis, TDP‐43, VEGF

## Abstract

**Background:**

This study focused on the mechanisms where icariin inhibited chondrocyte apoptosis and angiogenesis by regulating the TDP‐43 signaling pathway.

**Methods:**

A rat osteoarthritis (OA) model was established by collagenase injection. Histological examination of the articular cartilage and synovial tissue was performed 6 weeks after operation. Cartilage cell line overexpressing TDP‐43 and mesenchymal stem cell line (TDP43‐MSCs) of the rat *TDP43* gene were established.

**Results:**

In OA rats transplanted with TDP43‐mMSCs, TDP43 was highly expressed in chondrocytes (TDP43‐HC), while TDP43 expression was low in HC and MSCs‐HC (*p *<* *0.05). After the intervention of MSCs‐TDP43, high expression of TDP43 induced the apoptosis and death of chondrocytes. After the addition of icariin, late apoptosis and death of TDP43‐HC were significantly attenuated. Apoptosis and death of HC, MSCs‐HC, and TDP43‐HC cells were effectively controlled with icariin, and no apparent cell death was found. ELISA showed that the VEGF and HIF‐1 alpha were significantly higher in the rat OA model than the normal control rats.

**Conclusion:**

TDP43‐MSC transplantation interfered with the expression of TDP43 in the articular chondrocytes of OA rats, which may impact on inducing apoptosis of chondrocytes as well as inhibiting the proliferation of chondrocytes. Additionally, TDP43‐MSCs appeared to promote the formation of neovascularization in the synovial tissue, which could be significantly attenuated by icariin.

## 1 INTRODUCTION

Osteoarthritis (OA) is a common teratogenic disease of the skeletal system. The articular cartilage is the first and most invasive site of osteoarthritic lesions, and chondrocytes are the only cells that synthesize cartilage matrix within the articular cartilage. Studies have shown that there is an internal regulation system mediated by chondrocytes in the articular cartilage, which controls the occurrence and remodeling of the cartilage tissue, the stability of the internal environment, and pathophysiological processes such as wound repair (Arimoto‐Matsuzaki, Saito, & Takekawa, 2016; Huang et al., 2017; Vega et al., 2015). In OA, various pathological factors act directly or indirectly on chondrocytes, which regulate the dynamic equilibrium between cartilage matrix synthesis and degradation in the direction of degradation, and ultimately leads to the destruction of cartilage matrix (Hindle et al., 2016; Hwang & Kim, 2015; Yeung, Zhang, Wang, Yan, & Chan, 2018). How to effectively overcome the destruction of the internal control system of the articular cartilage is currently a hot and difficult issue. At present, reports have broadly confirmed that signaling pathways that participate in and regulate cell proliferation, differentiation, and inflammatory responses play an important role in the development of articular cartilage and in the occurrence and development of OA. Therefore, signaling pathways have been developed. It is possible to identify the intrinsic factors that destroy the internal control system of articular cartilage (Collins et al., 2015). OA belongs to the category of “deficiency syndrome” and “bone sputum” in traditional Chinese medicine. The deficiency of kidney essence is the fundamental internal cause of the disease, and the kidney‐deficiency blood stasis runs through the entire pathological process of OA. In this study, we establishes a rat OA model using TDP‐43 high‐expressing chondrocytes and collagenase to explore the effect of Chinese herb and icariin on TDP‐43 signaling in chondrocyte lesion, and chondrocyte apoptosis. In this context, we highlighted recent work describing the death and neovascularization, and analysis of the role of kidney tonic icariin on this molecular mechanism, to further understand the molecular mechanism whereby the Chinese herbal medicine reverses OA, and to provide a theory of traditional Chinese medicine in terms of prevention and treatment of OA.

## 2 MATERIALS AND METHODS

### 2.1 Materials

#### 2.1.1 Animals

SPF grade healthy and adult male Sprague‐Dawley rats (batch number: 00000) purchased from the Laboratory Animal Center of Medical University were used for standard breeding under the standard conditions with the same age and body weight of about 220–260 g under normal conditions for standard feeding. All animal experiments in this study were performed in accordance with the Guideline for the Management and Use of Laboratory Animal Revisions, revised by the Committee of Experimental Animals of the United States of America in 2012. During the study, researchers reduced the number of animals they used and their pain as low as possible.

#### 2.1.2 Major equipment and reagents

The following major equipment and reagents were used for the current study: Animal surgical instruments (Shenzhen), SIEMENS automatic biochemical analyzer (Tai Lin Orient Commerce Co., Ltd., Beijing), multi‐functional enzyme scale (Fisher, USA), Bole high current electrophoresis instrument (Beijing), spectrophotometer (Fisher company) SIGMA desktop high speed centrifuge (Sigma, Germany), tissue homogenizer (IKA, Germany); NIKON inverted phase contrast fluorescence microscope (Nikon, Japan), IKA constant temperature rocking platform (IKA, Germany), and electronic analytical balance (Mettle Toledo International Trade Co., Ltd., Switzerland).

### 2.2 Methods

#### 2.2.1 A rat OA model was established by injection of collagenase

Sixty Sprague‐Dawley rats were used. Collagenase (2.0 mg) was injected into the knee joint cavity of the hind limbs of rats to observe changes in the inflammatory response of the knee joints in rats. Histological examination of rat knee cartilage and synovial tissue was also carried out.

#### 2.2.2 Construction of TDP‐43 Overxpressing Chondrocyte Lines

Plasmids containing the full length *TDP‐43* gene and chondrocytes were obtained: the *TDP‐43* plasmid was sequenced. The project group preserved the chondrocyte line HC‐alpha, and constructed the *TDP43* lentivirus with the green fluorescence (*GFP*) reporter gene: the obtained full clone plasmid and auxiliary of the *TDP43* gene. Plasmids and the lentivirus packaging system were co‐transfected into 293T cells and the gene was obtained by endonuclease restriction 48 hr after the collection of lentivirus particles in the supernatant. The gene was purified, concentrated and sequenced and identified. HC‐alpha cells were infected with TDP43 lentiviruses. After cell lysis, isolation and extraction of total miRNA, TDP43 expression was verified by fluorescence PCR.

### 2.3 Establishment of *TDP43* overexpressing rat mesenchymal stem cell line (TDP43‐MSCs)

The MSCs were isolated and cultured from rat embryos. Cells were separated and purified using flow cytometry. Cell proliferation was identified in terms of growth curve, doubling time, and cell cycle, etc.; mesenchymal stem cell phenotype was identified through fluorescence PCR, flow cytometry, and immunocytochemistry.

### 2.4 Transplantation of TDP43‐mMSCs in the treatment of OA rats

OA rats were randomly divided into three groups, 10 in each group. Group A: simple MSCs were inoculated via the rat tail vein at 1–3 × 10^6^/rat in 500 μl; Group B: TDP43‐mMSCs were inoculated via the rat tail vein at 1–3 × 10^6^/rat in 500 μl; group C was the blank control group, and an equivalent volume of a fixed solution was injected in the same manner as group A and B.

### 2.5 Effects of icariin on TDP‐43 signaling

Based on TDP‐43 overexpressing chondrocyte cell line and TDP‐43‐mMSC inoculation in OA rats at different doses (10, 20, 40 ng/ml), the molecular effects of Chinese herb icariin on TDP‐43 signaling, including chondrocyte apoptosis and neovascularization were measured and analyzed.

#### 2.5.1 Apoptotic cell assays

Materials were obtained by extracting cells from the enzyme‐digested tissue. The tissues were rinsed with phosphate buffered saline (PBS), soaked in a Petri dish containing PBS, cut into pieces, and placed in a test tube. Three milliliters of PBS and 0.1% collagenase were added and the tissues were digested at 37°C for 48 hr. The tissue homogenate was clarified by centrifugation at 1,000 rpm for 10 to 15 minutes for two or three times and the pellets were saved. The number of live cells was 10^4^–10^6^/mm^3^. Tissue apoptotic cells were examined by annexin V/propidium iodide staining and flow cytometry.

### 2.6 Determination of VEGF and HIF‐1α in peripheral blood and joint tissues of OA rats

The levels of VEGF and HIF‐1α in peripheral blood and joint tissues of OA rats were determined by ELISA.

### 2.7 Statistical analysis

Data were expressed as mean ± standard deviation (ANOVA) and the difference was statistically significant between groups (*p *<* *0.05). SPSS19.0 statistical software was utilized for statistical analysis.

## 3 RESULTS

### 3.1 Expression of *TDP43* in HC, MSCs‐HC, and TDP43‐HC

**Figure 1:**
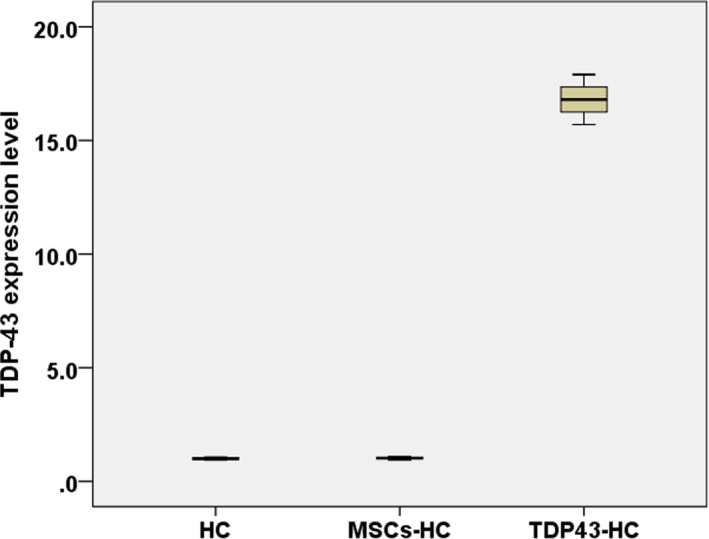
Fluorescent quantitative PCR assays of *TDP43* gene in HC, MSCs‐HC, and TDP43‐HC

*TDP43* expression in OA rat chondrocytes (TDP43‐HC) with TDP43‐mMSCs transplantation as well as the TDP43 expression in OA model rat chondrocytes (HC) and mesenchymal stem cells in OA model rat chondrocytes (MSCs‐HC) are presented in Figure 1. TDP43 was overexpressed in TDP43‐HC. However, TDP43 was at low levels in both HC and MSCs‐HC (*p* < 0.05).

### 3.2 Apoptosis analysis of icariin before and after HC, MSCs‐HC, and TDP43‐HC in Rats

**Figure 2:**
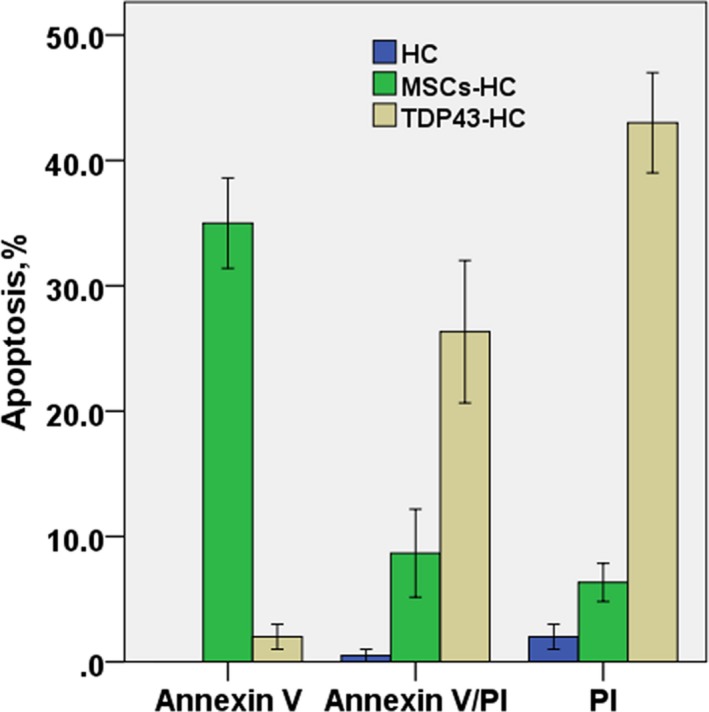
Analysis of the positive rate of apoptosis in rat HC, MSCs‐HC, and TDP43‐HC cultured without icariin for 7 days

HC, MSCs‐HC, and TDP43‐HC were cultured for 7 days without icariin, and stained with annexin V FITC and propidium iodide and examined by flow cytometry. TDP43‐HC showed late apoptosis (26.67% in the annexin V/propidium iodide double positive rate) and cell death (42.33%). A large number of apoptotic and dead MSCs‐HC were detected, with early apoptosis of cells (35.22% in Annexin V positive rate), while there were only 7.01% late apoptotic and dead cells. Early and late apoptosis and death of chondrocytes were very rare in the normal control group. The results showed that after treatment with MSCs‐TDP43, TDP43 overexpression induced the apoptosis and death of chondrocytes (Figure 2).

**Figure 3:**
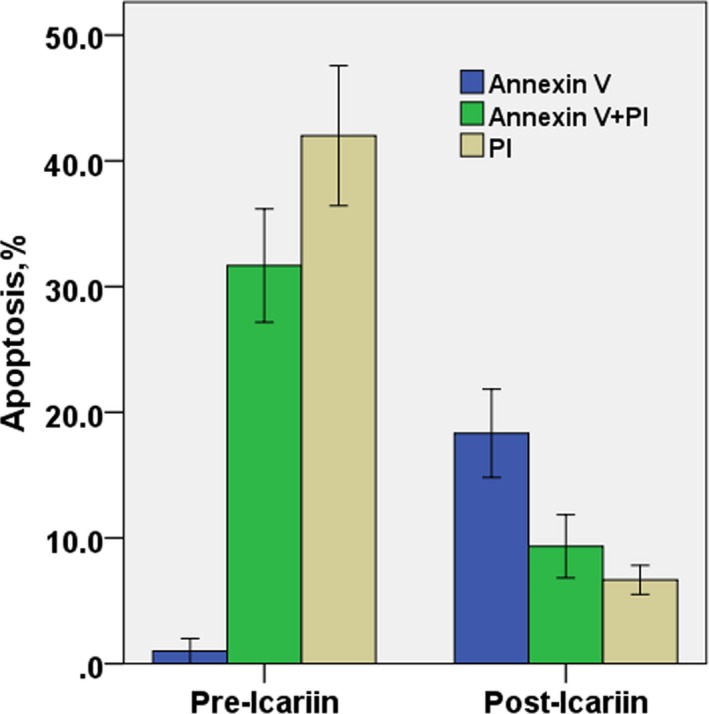
Flow cytometric analysis of the positive rate of apoptotic cells by annexin V and propidium staining after rat TDP43‐HC was added with icariin for 7 days

**Figure 4:**
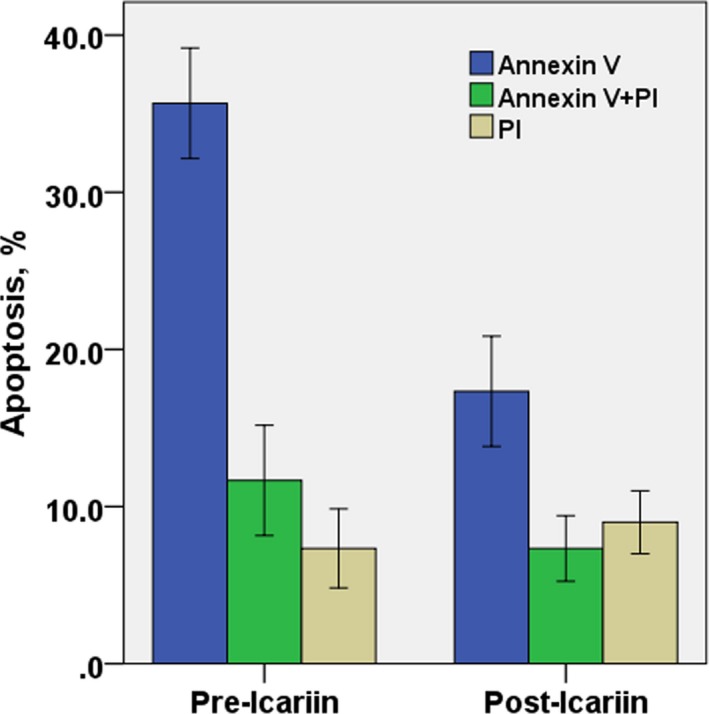
Flow cytometric analysis of the positive rate of apoptotic cells by annexin V and propidium staining after rat MSCs‐HC was added with icariin for 7 days

**Figure 5:**
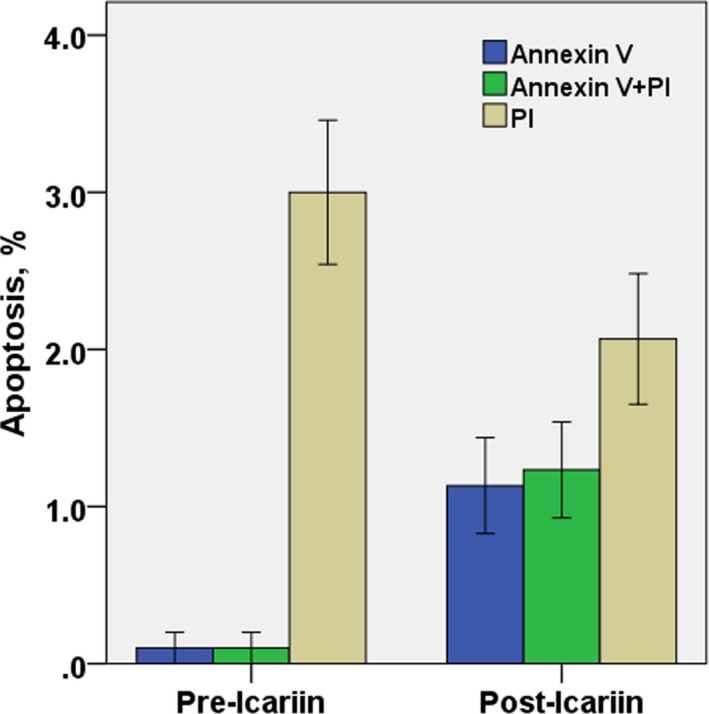
Flow cytometric analysis of the positive rate of apoptotic cells by annexin V and propidium staining after rat HC was added with icariin for 7 days

HC, MSCs‐HC, and TDP43‐HC were cultured with icariin for 7 days, and stained with annexin V FITC and propidium iodide and examined by flow cytometry. TDP43‐HC showed early apoptosis (19.67%annexin V positive cells) and cell death (8.67% annexin V FITC and propidium iodide positive cells) cell death. The chondrocytes did not undergo significant apoptosis and death (only 4.72% propidium iodide positive cells). However, MSCs‐HC showed early apoptosis of cells (17.43% annexin V positive cells), and that late apoptosis and death rate were lower than 10%, and early and late apoptosis and death of chondrocytes in the normal control group were 10%, which was rare. The results showed that after the intervention of MSCs‐TDP43, TDP43 overexpression induced apoptosis and death of chondrocytes. Furthermore, after the addition of icariin to chondrocytes, late apoptosis of TDP43‐HC (annexin V FITC and propidium iodide positive cells) and the death phenomenon (propidium iodide positive cells) were significantly reduced. The effect of Icariin on HC, MSCs‐HC, TDP43‐HC cells in late death and death were effectively controlled by icariin. No apparent cell death occurred (Figures 3, 4, and 5).

### 3.3 Effects of different doses of icariin on the proliferation of HC, MSCs‐HC and TDP43‐HC cells

**Figure 6:**
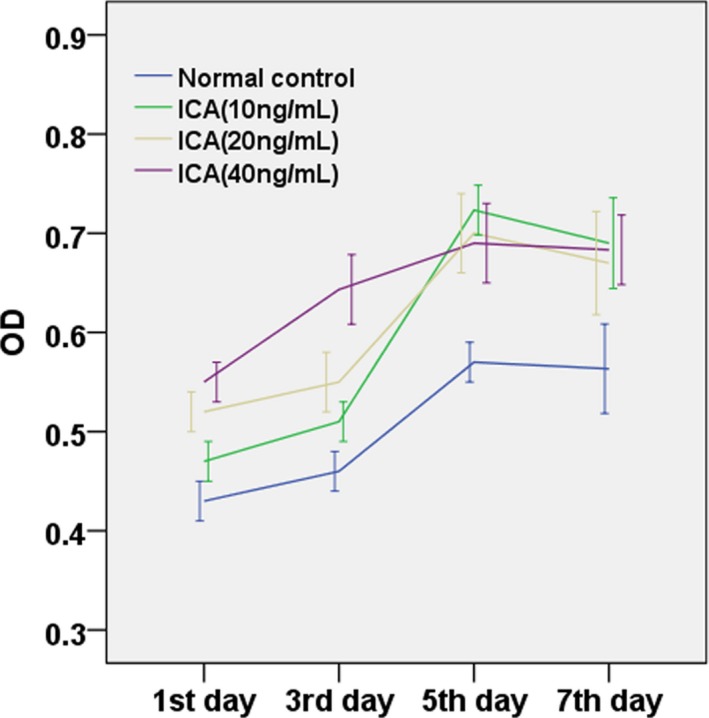
Growth curves of rat HC support culture in the presence of different doses of icariin

The tetrazolium colorimetric assay (MTT) was used to detect the growth curve of icariin on rat knee HCs. Rat HCs were cultured from day 1 to day 7 among the normal control group and different doses of icariin HCs (Figure 6). The trend of the growth curve was highly consistent; the effects of different doses of icariin on the proliferation of HC varied with culture time. Cellular growth was slow from day 1 to day 3, and the growth reached a peak from day 3 to day 5, followed by a decreasing trend in cellular growth. Icariin at 10 ng/ml showed the most significant cell proliferation trend from day 3 to day 5 when compared to the normal control group and other groups treated with different doses of icariin. In contrast, the Yang Qi group had no significant cell proliferation d from day 3 to day 5 of culture, and even had a reduced trend in the proliferation from day 5 to day 7.

**Figure 7:**
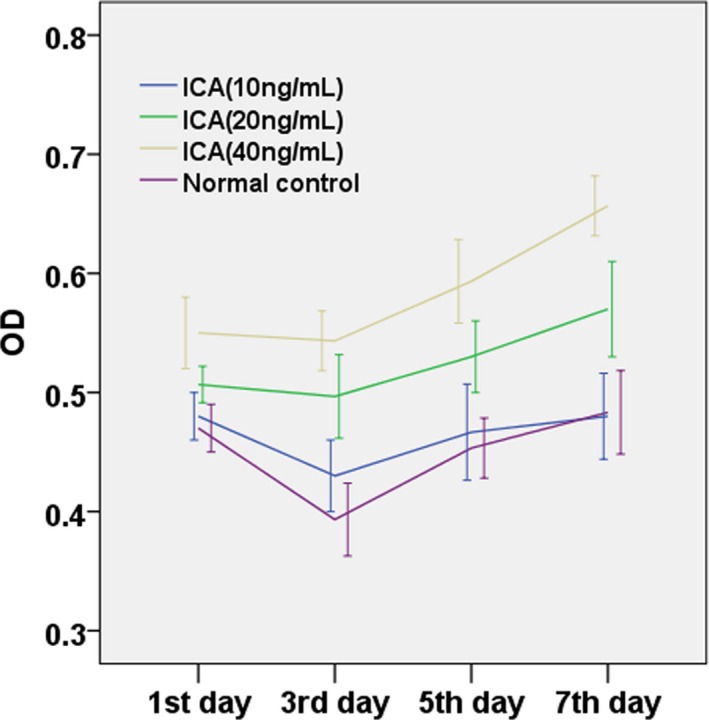
Growth curves of rat MSCs‐HC support culture in the presence of different doses of icariin

MTT assay was used to determine the growth curve of MSCs‐HC treated with icariin in knee joints in rats. Growth of MSCs‐HC in the normal control group and that treated with different doses of icariin from day 1 to day 7 are shown in Figure 7. The trend of the curves tended to be consistent; the effect of different doses of icariin on the proliferation of MSCs‐HC varied with culture time, and cell growth was reduced from day 1 to day 3 of culture, and peaked at day 3 to day 7. The proliferation of cells treated with icariin at a dose of 40 ng/ml increased significantly from day 3 to day 5 compared with other groups. The icariin group at a dose of 10 ng/ml was the closest to the normal control group. Cellular proliferation from day 3 to day 5 of culture period was remarkable, and slowed down from day 5 to day 7.

**Figure 8:**
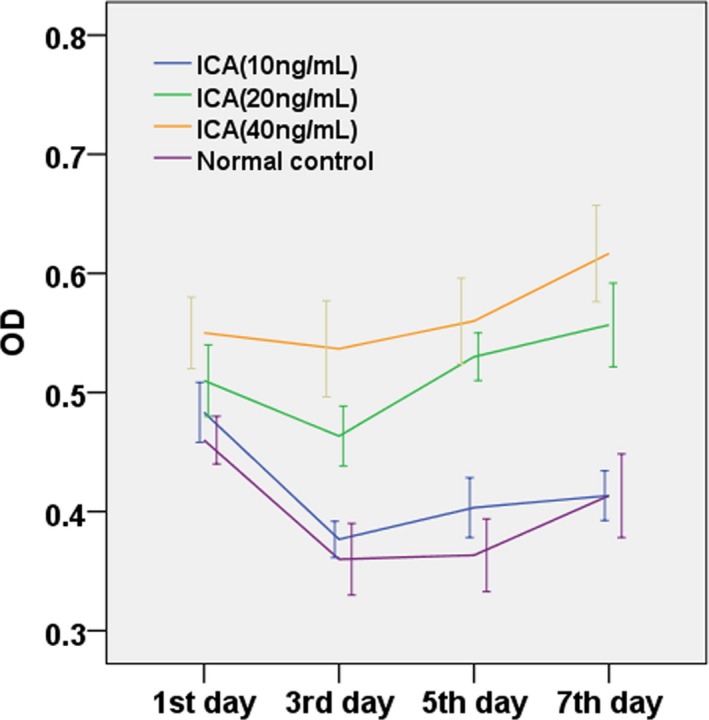
Growth curves of rat TDP43‐HC supporting culture in the presence of different doses of icariin

The results of MTT assays for the growth curve of icariin on TDP43‐HC in rat knee joints showed the growth of MSCs‐HC in the normal control group and different doses of icariin from day 1 to day 7 after rat TDP43‐HC culture (Figure 8). The trend of the curves tended to be consistent; the effects with different doses of icariin on the proliferation of TDP43‐HC varied with culture time. Cellular growth slowed down from day 1 to day 3 among the four groups. The proliferation of cells treated with 20 ng/ml increased remarkably from day 3 to day 5 compared with that of the normal control group and other dose groups. Icariin at 10 ng/ml was the closest to the normal control group from day 3 to day 5 of culture. Significant cellular proliferation was found in the results.

### 3.4 Effect of different doses of icariin on the expression of VEGF and HIF‐la in rats

Synovial tissue ELISA in rats showed that VEGF and HIF‐1α in OA rats were significantly higher than those in normal control rats. VEGF and HIF‐1α expression in the synovial tissue was increased with different doses of icariin. The expression levels of VEGF and HIF‐1α decreased gradually, and there were significant differences between the groups (*p *<* *0.05) (Table 1).

**Table 1:** Expression level of VEGF and HIF‐1α in the CIA synvium

Group	VEGF	HIF‐1α
Normal control	17.64 ± 3.57^##^	13.85 ± 1.51^##^
OA model	168.43 ± 11.24**	54.28 ± 2.69**
ICA(10 ng/ml)	118.35 ± 9.21*^#^	48.52 ± 3.65*^#^
ICA(20 ng/ml)	69.58 ± 5.81*^#^	33.27 ± 2.18*^#^
ICA(40 ng/ml)	37.62 ± 3.64*^#^	18.62 ± 1.71**

**Compared to the control group, *P* < 0.05; ^##^compared to the OA model group *P* ＜ 0.05; ^*#^compared to control and OA group, *P* < 0.05.

Rat peripheral blood ELISA showed that VEGF and HIF‐1α expression in OA rats was significantly higher than that of the normal control rats. VEGF levels in the peripheral blood increased with different doses of icariin. Moreover, the expression level of HIF‐1α decreased gradually, and the difference between the groups was statistically significant (*p* < 0.05) (Table 2).

**Table 2:** Expression level of VEG and HIF‐1α in serum

Group	VEGF	HIF‐1α
Normal control	10.43 ± 1.14^##^	3.85 ± 1.01^##^
OA model	17.86 ± 1.42**	5.27 ± 0.89**
ICA(10 ng/ml)	16.34 ± 1.33^##^	4.67 ± 0.75*^#^
ICA(20 ng/ml)	14.27 ± 1.25*^#^	3.29 ± 0.98^##^
ICA(40 ng/ml)	10.55 ± 1.12**	2.77 ± 0.61**

**Compared to the control group, *P* < 0.05; ^##^compared to the OA model group *P* ＜ 0.05; ^*#^compared to control and OA group, *P* < 0.05.

## 4 DISCUSSION

OA occurs in an inflammatory environment involving an immune response and a stress response due to lack of nutrients (Wolfstadt, Cole, Ogilvie‐Harris, Viswanathan, & Chahal, 2015). This inflammatory environment is persistent and can trigger apoptosis and the formation of new blood vessels. Therefore, finding a way to suppress inflammatory condition is of great importance. The inflammatory environment and stress response involving immunity may be effective methods for the treatment of OA, and has also become a hotspot and key issue for the study of the development of OA (Maumus et al., 2016; Zhou et al., 2015). OA is often accompanied by abnormal signal transduction in secretion and activation of cytokines and inflammatory cytokines, suggesting that signal transduction may play an important role in the development and progression of OA. In recent years, studies have shown that signaling pathways involved in and modulating cell proliferation, differentiation, apoptosis, and inflammatory responses play a very crucial part in the development of bone and joints as well as in the occurrence and development of OA.

MSCs can effectively exert their immunosuppressive and anti‐inflammatory effects, especially in the treatment of OA; the immunosuppressive and anti‐inflammatory functions of MSCs have been confirmed in both in vitro and in vivo experiments. A large number of studies have confirmed that MSCs in vivo can regulate the immune responsiveness of dendritic cells, B cells, and T cells, inhibit NK cell proliferation, cytokine secretion, and cytotoxicity, and play an anti‐inflammatory role (Dalen et al., 2015; Rai, Dilisio, Dietz, & Agrawal, 2017).

This study attempted to study the possibility of stem cell treatment of OA from TDP‐43, which affects signal transduction, and is helpful to clarify the mechanism of OA in stem cell therapy. According to the theory of “kidney competent bone marrow production” in traditional Chinese medicine, modern medical studies on bone marrow mesenchymal stem cells (BMSCs) in relation to “kidney competent bone”, and kidney tonifying Chinese medicine have the function of directional differentiation to BMSCs. Traditional Chinese medicine treatment of OA in the kidney and activating blood is the basic treatment, which is to dehumidify the wind, remove the cold, and eliminate and treat the symptoms. Icariin is a commonly used traditional Chinese medicine for OA in many kidney tonifying herbs. It is also one of the most studied Chinese medicines worldwide. It has the function of invigorating kidney and Yang, removing wind and dehumidification. In this study, it may affect the biological characteristics of stem cells, promote the proliferation and differentiation of BMSCs, and achieve good effect in the treatment of OA by acting on the signal pathway of OA.

Studies have found that cytokines rely on the activation of two signal transduction pathways involved in the development and progression of OA, namely JNK (Berenbaum, Griffin, & Liu‐Bryan, 2017; Xia et al., 2015) and p38MAPK (Zhou et al., 2015). Both signaling pathways can activate the important activation substrates AP‐1 protein ATF‐2, and NF‐κB, etc. to promote apoptosis of the visual cell, and the p38MAPK signaling pathway is an upstream regulatory kinase of VEGF that may regulate VEGF expression. In response to endoplasmic reticulum stress, JNK and p38MAPK signaling pathways and apoptosis are inhibited at the same time (Xia et al., 2015), thus increasing the expression of TDP‐43 that promotes the formation of SGs, thereby blocking cytokine‐dependent cell signaling pathway and leading to the occurrence and development of OA. It has been experimentally confirmed that VEGF stimulates the generation and enhancement of new blood vessels by activating MEK1/2 and p38 of the MAPK signaling pathway, and the use of specific blocking agents to block the above two pathways can inhibit neovascularization (Li et al., 2017). More and more studies have found that MAPK, especially the p38/MAPK pathway, is related to the promotion of VEGF production and neovascularization (Yu & Kim, 2015).

Apoptosis of chondrocytes may be an important cause of OA. It is generally believed that the p38 signaling pathway is one of the upstream signaling pathways of articular chondrocyte apoptosis. Both NO and IL‐β can induce apoptosis of chondrocytes by activating p38. Blocking p38 can significantly block OA cartilage cell apoptosis. The activation of p38 can promote the apoptosis of articular hyaline cartilage cells and inhibit the apoptosis of fibrocartilage cells. Recent studies have found that the expression of 35‐kDa isoform (TDP‐43/p35) of TDP‐43 can promote the formation of SGs. MSCs can be used as a vector for gene therapy and carry the *TDP‐43* gene into damaged cartilage tissue to reduce vascular endothelial cells through permeabilization, inhibition of inflammatory factors and stress‐mediated apoptosis pathways, and synergy with stem cell therapy (Higashi et al., 2013).

Occurrence of OA is based on the deficiency of kidney essence, kidney deficiency, and blood stasis. Blood stasis is the pathological manifestation of OA, and kidney deficiency and blood stasis go through the entire pathological process of OA. Chinese medicine treats OA for Bushen Huoxue as the basic governing method, through which the combination of kidney and blood circulation can make kidney strong, smoothen meridian, prevent the occurrence and development of lesions. In many kidney herbs, icariin is the most commonly used traditional Chinese medicine for the treatment of OA. It can tonify the kidneys, strengthen yang, and remove wind and dampness. Studies have shown that icariin's own active ingredients can promote the proliferation and differentiation of osteoblasts, promote the body to produce factors that contribute to the growth of osteoblasts and promote the proliferation and differentiation of osteoblasts, as well as inhibit osteoclasts. Additionally, icariin can activate the ERK/MAPK and P38/MAPK signaling pathways in osteoblasts and promote the expression of nuclear transcription factor Cbfa1 in osteoblasts. It has been reported recently (Shantanu et al., 2017; Zhao et al., 2015) that icariin did not promote cell proliferation, but a higher concentration of icariin inhibited cell proliferation. A certain amount of icariin could highly promote osteogenic differentiation of primary cultured BMSCs.

Taken together, TDP43‐MSC transplantation interfered with the expression of TDP43 in the articular chondrocytes of OA rats, which may impact on induction of apoptosis of chondrocytes as well as inhibiting the proliferation of chondrocytes. Furthermore, TDP43‐MSCs have the effect of promoting the formation of new blood vessels in the synovial tissue of joints. We also demonstrate that icariin can inhibit the above process significantly.

## CONFLICT OF INTEREST

The authors declare there is no conflict of interest involved in this study.
